# Efficient Markets and Contingent Claims Valuation: An Information Theoretic Approach

**DOI:** 10.3390/e22111283

**Published:** 2020-11-12

**Authors:** Jussi Lindgren

**Affiliations:** Department of Mathematics and Systems Analysis, Aalto University, 02150 Espoo, Finland; jussi.lindgren@aalto.fi

**Keywords:** information theory, financial markets, entropy, stochastic optimal control, options pricing, Black–Scholes equation, Burgers equation, free energy

## Abstract

This research article shows how the pricing of derivative securities can be seen from the context of stochastic optimal control theory and information theory. The financial market is seen as an information processing system, which optimizes an information functional. An optimization problem is constructed, for which the linearized Hamilton–Jacobi–Bellman equation is the Black–Scholes pricing equation for financial derivatives. The model suggests that one can define a reasonable Hamiltonian for the financial market, which results in an optimal transport equation for the market drift. It is shown that in such a framework, which supports Black–Scholes pricing, the market drift obeys a backwards Burgers equation and that the market reaches a thermodynamical equilibrium, which minimizes the free energy and maximizes entropy.

## 1. Introduction

The pricing of contingent claims or financial derivatives was revolutionized in 1973, when the Black–Scholes pricing model was presented [1]. The celebrated Black–Scholes pricing equation is a linear partial differential equation (PDE), which describes the evolution of the value of the financial derivative with some boundary conditions, depending on the assumptions. The boundary conditions are determined from the contractual details of the financial instrument. For example, for European call/put options, an explicit pricing formula is available. Options and other derivatives are used daily in the financial markets, as they can be used in speculating on or insuring against financial risks related to economic activities. For example, options give the holder the right, but not the obligation, to buy or sell an instrument at a specified time at a specified price. Such derivative instruments are paramount for international trade to flow smoothly and for other financial risk management and hedging purposes. The prices of contingent claims can be technically obtained by solving the corresponding pricing PDE using discretization and numerical methods (finite-difference) or Monte Carlo simulations (stochastic representation of PDE solutions, Feynman–Kac). It should be noted that option theory can be used also to value uncertain real investments; see, for example, [2]. Even though, e.g., the pricing of options is a well-established field of research, less focus has been put on the properties of the financial market itself that support no-arbitrage pricing of financial derivatives, but see, for example, [3]. For a good introduction to mathematical finance and valuation, see, e.g., [4].

The approach, in which the derivation of the Black–Scholes PDE is economically speaking easiest to understand, is probably the delta-hedging approach, as in [1], where one creates a replicating portfolio of the underlying asset and the risk-free asset and perfectly replicates the value of the financial derivative. Using the principle of no-arbitrage and the tools of stochastic analysis, one can then show that the value of the contingent claim indeed in complete markets with no-arbitrage must obey the Black–Scholes equation. Key technical assumptions include in particular the existence of a risk-free asset, the possibility to re-balance the hedged portfolio continuously, no transactions costs, the availability of short-selling, and the infinite divisibility of the underlying asset. There are other approaches such as risk-neutral pricing [5], but the original delta-hedging approach is probably the most intuitive one. The Black–Scholes PDE is the following: Consider a derivative security that yields a payoff of Y(S(T)) where Y:R→R is a continuous function and *S* is the random spot price of the underlying asset. The derivative security payoff at time τ<T is C(S(τ),τ). The corresponding Black–Scholes partial differential equation for the value of the derivative security is given (see [4]) by:

(1) $$C{\left(s,\tau \right)}_{\tau }=-rsC{\left(s,\tau \right)}_{s}-\frac{1}{2}\sigma^{2}s^{2}C{\left(s,\tau \right)}_{ss}+rC\left(s,\tau \right),$$

for $\left(s,\tau \right)\in R_{+}\times \left[0,T\right)$, with the boundary condition C(s,T)=Y(s). The riskless rate of return is denoted by r>0, and the volatility is σ>0. Subscripts refer to partial differentiation throughout the study. By using the fact that $S=e^{X}$, we can equivalently write the Black–Scholes equation (see, e.g., [6]) for the log-price:

(2) $$C{\left(x,\tau \right)}_{\tau }=\left(\frac{1}{2}\sigma^{2}-r\right)C{\left(x,\tau \right)}_{x}-\frac{1}{2}\sigma^{2}C{\left(x,\tau \right)}_{xx}+rC\left(x,\tau \right),$$

which is a linear, constant coefficient PDE.

In this study, a novel approach is taken to derive the Black–Scholes pricing PDE. A stochastic optimal control problem is developed, where the value of the contingent claim is shown to reflect the total expected information generated by the financial market. The model is inspired by the information theory of Claude Shannon [7] through the concept of entropy as expected information, and therefore, it naturally links it to price processes in financial markets. One natural benefit of this approach is that one can deduce directly a transport equation for the drift of the log-price of the underlying asset, which should be satisfied in such complete financial markets, when there is no arbitrage. Moreover, using the tools of stochastic processes, we can study the “thermodynamic equilibrium” of the financial market.

There has been little research on such implied transport equations for the drift or the market risk premium, but see, for example, [3,8]. In these cited articles, it is shown by arguing via certain path-independence arguments and utilizing Girsanov’s theorem (change of measure) that the market risk premium must obey the Burgers equation, which can be used as a simple model for turbulent fluid flow and traffic flows; see, for example, [9]. Indeed, the transport equation derived in the present study is the backwards Burgers equation. Finally, we can study the relaxation of the market towards equilibrium market conditions, where the log-price distribution reaches a stationary state. It is shown that this relaxation of the market towards equilibrium is a spontaneous process in which the “free energy” is minimized, and in the equilibrium, the entropy is maximized. A financial market supporting no-arbitrage pricing seems therefore to obey a form of the second law of thermodynamics. Deducing dynamics or PDEs for the drift and/or the risk premia could be potentially useful in the context of portfolio management; for example, see [8].

Entropy and information theory have been utilized in financial theory recently, for example in [10,11,12]. Stochastic optimal control has been used rarely in options pricing, but see the seminal paper [13]. Recently, an entropic approach to options pricing was taken in [14], where the authors utilized Jaynes’ approach [15] of maximum entropy. It can be argued that there is a direct link here to that approach via the Gibbs distribution, as the stationary Gibbs distribution is known to maximize entropy when the expected energy is fixed.

The efficient market hypothesis [16] and information theory have been considered together, especially from the point of view of Kolmogorov complexity; see [17,18]. As the efficient market hypothesis states loosely that properly anticipated prices fluctuate randomly due to the assumption that all relevant information is already taken into account in prices, it seems rather natural indeed to consider the market prices from an algorithmic complexity perspective. The Kolmogorov complexity of a string is the binary length of the shortest program run in a universal Turing machine that produces the output string. This definition means that a truly random sequence (such as maybe price sequences in the financial market) is incompressible in some sense; there exists no short algorithm to generate it. Kolmogorov complexity and algorithmic information theory in general consider discrete random variables in time, whereas in the present work, the random variables evolve continuously in time. However, what is common with the present study and algorithmic complexity approaches is that we need to quantify and measure information and randomness in terms of the price processes. The measure of information in the present work is a certain conditional expectation of cumulative changes in the drift of a price process.

## 2. The Model

### 2.1. The Information Content of Financial Markets

We have an asset, such as a common stock, whose log-price X(t) is assumed to be evolving according to the stochastic differential equation (SDE):

(3) dX(t)=μ(X(t),t)dt+σdW(t),

where W(t) is standard Brownian motion, μ is the drift of the process or the instantaneous expected rate of return, and the constant σ is the volatility or the diffusion coefficient describing the amplitude of the noise. The price of the asset, S(t), at time *t* is in other words given by $S\left(t\right)=e^{X(t)}$.

Consider what is to be understood as the information content related to a price process. We assume that the log-price process X(t) follows an Ito diffusion with some drift μ and diffusion coefficient σ. The efficient market hypothesis states that all relevant information is already represented in the price of the asset [16]; therefore, any new and relevant information will most likely change the price to a measurable extent, if the market is close to efficient. We therefore expect that the local market information content *I* is a function of the local drift μ of the log-price process, so that a local information function can be defined, and it is of the general form I=I(μ). If this is the case, we expect that the total amount of information generated by the financial market within the time period [τ,T] is given by:

(4) $$E_{\tau x}\left(\int_{\tau }^{T}I\left(\mu \right)dt\right).$$

The functional above is an expectation with conditioning on the initial state X(τ)=x. This is the functional the market “wants” to minimize, in order to be as efficient as possible; in other words, the log-price drift should accommodate any new available information fully, efficiently, and without delay. We assume a simple quadratic functional form for the information function *I*. This assumption comes from the reasoning that we interpret local information as the squared deviation of the drift from some constant *b* (*c* is included to fix the origin of the information measure):

(5) $$I\left(\mu \right)={\left(a\mu (X(t))-b\right)}^{2}+c=\frac{1}{2}\alpha \mu^{2}+\beta \mu +\gamma ,$$

with some constants a,b,c,α,β,γ∈R. The quadratic cost function implies that it “pays off” for the market to adapt the price of the instrument immediately when new relevant market information is common knowledge; in other words, large deviations in the drift are penalized quadratically. The quadratic local information function can be argued to be a reasonable starting point, as utilizing Occam’s razor, it is the simplest function that has desirable symmetry and is differentiable. Moreover, any information function is locally well approximated by a quadratic approximation (Taylor’s theorem). Quadratic cost functions are common in engineering and physics perhaps for these reasons. Technically, the choice of a quadratic cost function makes the Hamiltonian quadratic, which also allows one to find solutions due to the existence of maxima or minima. As can be seen later on, the quadratic Hamiltonian leads to the Burgers equation due to the quadratic nonlinearity in the Hamilton–Jacobi–Bellman equation.

### 2.2. The Stochastic Optimal Control Problem for the Financial Market

We follow the notation used in [19], which is an excellent source on stochastic optimal control and applications. Assume now that the financial market effectiveness is reflected in the total amount of information generated by the price process. Intuitively, the drift should be as constant as possible. Moreover, it is assumed that the financial market also minimizes the expected value of a penalty function g(X(T)) at terminal time T>t. Note that the penalty function at the terminal time depends only on the price of the underlying asset at time *T*. The penalty function can be seen to represent a target for the log-price, and the accumulating information during the time [τ,T] is a cost incurred to transport the log-price. The optimization scheme is therefore:

(6) $$\min_{\mu }E_{\tau x}\left(\int_{\tau }^{T}I\left(\mu \right)dt+g\left(X\left(T\right)\right)\right),$$

subject to the market dynamics:

(7) dX(t)=μdt+σdW(t).

Note that the stochastic optimal control problem above resembles technically Merton’s optimal portfolio problem [20], where the consumer is maximizing her/his utility while allowing for investment in a portfolio consisting of risk-free and risky assets. In Merton’s model, the agent is a human being maximizing consumption welfare. In the present model, there is a finite horizon, and the market is collectively trying to be as “efficient as possible”; the cost function is related to information, whereas in Merton’s model, the agent is trying to maximize lifetime utility from consumption and investing optimally according to her/his risk aversion preferences. In physics and engineering, such similar optimal transport programs as the present model are common, where the system tries to minimize the spent energy whilst trying to get from point *A* to point *B*. It should also be noted that in physics, energy and information are essentially the same entity, and temperature links these two concepts together.

The dynamic programming equation for the value function J(x,τ) or the Hamilton–Jacobi–Bellman equation can be determined by first maximizing the Hamiltonian of the stochastic optimal control problem H with respect to the control μ; we assume that the value function is smooth, as is the case (see [19]),

(8) $$\max_{\mu }H=\left(-\mu J_{x}-I\left(\mu \right)\right).$$

The candidate $\mu_{*}$ for the optimal Markov policy is obtained using calculus:

(9) $$\mu_{*}=-\frac{1}{\alpha }J_{x}-\frac{\beta }{\alpha }.$$

To obtain a maximum for the Hamiltonian, we require that α>0, so that the Hamiltonian is concave in the control.

We then consider the transport equation for the value function J(x,τ). The Hamilton–Jacobi–Bellman equation is given using the optimal policy:

(10) $$J_{\tau }+\frac{1}{2}\sigma^{2}J_{xx}=H_{*}.$$

The maximized Hamiltonian is given by:

(11) $$H_{*}=\left(\frac{1}{\alpha }J_{x}+\frac{\beta }{\alpha }\right)J_{x}-\frac{1}{2}\alpha \mu_{*}^{2}-\beta \mu_{*}-\gamma ,$$

(12) $$H_{*}=\left(\frac{1}{\alpha }J_{x}+\frac{\beta }{\alpha }\right)J_{x}-\frac{1}{2}\alpha {\left(-\frac{1}{\alpha }J_{x}-\frac{\beta }{\alpha }\right)}^{2}-\beta \left(-\frac{1}{\alpha }J_{x}-\frac{\beta }{\alpha }\right)-\gamma ,$$

which, after simplifying, gives:

(13) $$H_{*}=\frac{1}{2\alpha }{\left(J_{x}\right)}^{2}+\frac{\beta }{\alpha }J_{x}+\frac{1}{2}\frac{\beta^{2}}{\alpha }-\gamma .$$

Next, we linearize the Hamilton–Jacobi–Bellman equation by using the Hopf–Cole transformation. Using now the substitution J=κlogC, κ∈R and assuming that the function C(x,τ) has smoothness at least $C\in C^{2,1}$, the Hamilton–Jacobi–Bellman equation in the present study is actually uniformly parabolic (see [19]), so that the equation possesses a unique smooth solution. We obtain:

(14) $$\kappa \frac{C_{\tau }}{C}+\frac{1}{2}\sigma^{2}\kappa \left(\frac{C_{xx}}{C}-{\left(\frac{C_{x}}{C}\right)}^{2}\right)=\frac{\kappa^{2}}{2\alpha }{\left(\frac{C_{x}}{C}\right)}^{2}+\frac{\kappa \beta }{\alpha }\frac{C_{x}}{C}+\frac{1}{2}\frac{\beta^{2}}{\alpha }-\gamma ,$$

In order to linearize the PDE, we need to have $-\frac{1}{2}\sigma^{2}\kappa =\frac{\kappa^{2}}{2\alpha }$. We choose κ=−1, and therefore, $\alpha =\frac{1}{\sigma^{2}}>0$.

(15) $$C_{\tau }+\frac{1}{2}\sigma^{2}C_{xx}=\frac{\beta }{\alpha }C_{x}+\left(\gamma -\frac{1}{2}\frac{\beta^{2}}{\alpha }\right)C.$$

### 2.3. Matching Coefficients

The linearized Hamilton–Jacobi–Bellman Equation (15) is exactly the Black–Scholes pricing Equation (2) for the log-price, when we identify the parameters of the model properly. The Black–Scholes equation for the log-price is:

(16) $$C_{\tau }+\frac{1}{2}\sigma^{2}C_{xx}=\left(\frac{1}{2}\sigma^{2}-r\right)C_{x}+rC.$$

Therefore, we require:

(17) $$\frac{\beta }{\alpha }=\left(\frac{1}{2}\sigma^{2}-r\right)$$

and:

(18) $$\left(\gamma -\frac{1}{2}\frac{\beta^{2}}{\alpha }\right)=r.$$

As we had already chosen $\alpha =\frac{1}{\sigma^{2}}$, we must have $\beta =\frac{1}{2}-\frac{r}{\sigma^{2}}$ and $\gamma =\frac{1}{2}\frac{\beta^{2}}{\alpha }+r$.

The terminal data for the linearized Hamilton–Jacobi–Bellman equation, that is for the Black–Scholes equation, are obtained by applying the same Hopf–Cole transformation to the penalty function g(X(T))=−logY(X(T)). Then, C(x,T)=Y(X(T)). As *Y* is the payoff of the contingent claim, the stochastic control problem aims to maximize the expected log-payoff of the contingent claim whilst minimizing the cumulative information-related cost.

## 3. The Transport Equation for the Market Drift

**Theorem** **1.** *The market supporting Black–Scholes pricing requires that the drift obeys the backwards Burgers equation.*

**Proof.**  Consider now again the derived Hamilton–Jacobi–Bellman transport Equation (10) for the value function J(x,τ). Differentiate partially once with respect to *x* and using the optimal policy $\mu_{*}=-\frac{1}{\alpha }J_{x}-\frac{\beta }{\alpha }$:

(19) $$J_{\tau x}+\frac{1}{2}\sigma^{2}J_{xxx}=\frac{1}{\alpha }J_{x}J_{xx}+\frac{\beta }{\alpha }J_{xx};$$

substituting the partial derivatives of the value function, we obtain the backwards Burgers equation:

(20) $$\mu_{\tau }+\mu \mu_{x}+\frac{1}{2}\sigma^{2}\mu_{xx}=0.$$

This completes the proof. Note that it is a nonlinear PDE. This nonlinear dynamics governs the drift or the instantaneous return of the complete and arbitrage-free market supporting the Black–Scholes pricing dynamics. The PDE must be solved backwards, derived from the original boundary condition J(X(T))=g(X(T)). □

## 4. The Free-Energy of the Market and Relaxation towards the Thermodynamical Equilibrium

As the Ito diffusion for the market supporting Black–Scholes pricing has a drift that is a gradient, consider the Fokker–Planck or Kolmogorov forward equation for the log-price process with the optimal drift:

(21) $$dX\left(t\right)=\left(-\frac{1}{\alpha }J_{x}-\frac{\beta }{\alpha }\right)dt+\sigma dW\left(t\right),$$

This optimal diffusion has a gradient structure having formally the potential function:

(22) $$V=\frac{J}{\alpha }+\frac{\beta }{\alpha }x.$$

Under mild technical conditions, this diffusion has a well-defined stationary distribution, which is the Gibbs distribution from statistical mechanics (see [21]):

(23) $$p_{\infty }=\frac{1}{Z}e^{-\frac{2}{\sigma^{2}}V},$$

where $Z=\int e^{-\frac{2}{\sigma^{2}}V}dx$ is the partition function or normalization constant ensuring that the total probability mass is normalized to unity. This $p_{\infty }$ is the stationary distribution for the log-price characterizing the “thermodynamic equilibrium” of the market. The existence of the stationary distribution requires some technical features from the value function; essentially, it has to grow sufficiently fast when x⟶±∞, and this guarantees that the potential is confining, the process is ergodic, and convergence to the Gibbs distribution is exponentially fast.

### Free-Energy and Relative Entropy Minimization

As was shown explicitly in [21], a free-energy functional F(ρ) exists, which is a Lyapunov function for these types of gradient diffusions, so that it decreases as time goes forward, $\frac{dF}{dt}\leq 0$. Such a free-energy functional is defined as:

(24) $$F\left(\rho \right)=\int V\rho dx+\frac{1}{2}\sigma^{2}\int \rho \log\rho dx+\frac{1}{2}\sigma^{2}\log Z,$$

where ρ is the transition probability density, which satisfies the Fokker–Planck equation governing the transition probability density for the log-price:

(25) $$\rho_{t}={\left(\rho \frac{\partial V}{\partial x}\right)}_{x}+\frac{1}{2}\sigma^{2}\rho_{xx}.$$

The free-energy functional is essentially the Kullback–Leibler distance of the transition density from the stationary density; it is also called the relative entropy [21]. As is well known, the Gibbs distribution is the maximum entropy distribution for a fixed energy [15].

## 5. Discussion and Conclusions

This research article demonstrates how the valuation of contingent claims in finance can be naturally seen from an information theoretic perspective. A natural stochastic optimal control program for minimizing the inefficiency of the financial market leads to a linearized Hamilton–Jacobi–Bellman equation, which is exactly the Black–Scholes pricing equation. The optimal transport for the instantaneous asset return is shown simultaneously to obey the backwards Burgers equation, and the system relaxes towards a thermodynamic equilibrium. The results support the earlier findings in [3], where a somewhat different argumentation based on path-independence led to similar Burgers equations for the market risk premium. If indeed a complete and arbitrage-free financial market that supports Black–Scholes pricing implies a nonlinear diffusion equation for the return, obviously, the results could be useful in portfolio management in predicting market returns by using econometric models, for example. Furthermore, the stochastic optimal control problem for the financial market can be seen as an operationalization of the efficient market hypothesis. According to the efficient market hypothesis, new information should be embedded in the market prices almost instantly. It is quite interesting that such an efficiency requirement for the market implies a nonlinear transport equation for the asset returns. As is well known, the Burgers equation can be used to model turbulent fluid flow.

The present article shows also the benefit of utilizing the methodology of physics in economics and finance, and indeed, econophysical research should be further encouraged. Continuous-time finance and options pricing have been also linked to quantum mechanics; see, for example, the recent book [6]. The present author concludes that there is indeed a methodological link between mathematical valuation of derivatives and quantum mechanics, and the methodological bridge is that of stochastic optimal control theory and diffusion theory. Recently, it has been shown that the wave equations of relativistic quantum mechanics can be derived from a stochastic optimal control approach, (see [22]) and that the Born rule and the Heisenberg uncertainty principle can be understood from this approach in a straightforward manner as well (see [23]).
